# Supplementary material for: Impact of the COVID-19 pandemic on neonatal admissions in a tertiary children’s hospital in southwest China: An interrupted time-series study
Source: PLoS One. 2022 Jan 13;17(1):e0262202. doi: 10.1371/journal.pone.0262202 (PMC8758080; doi:10.1371/journal.pone.0262202)
Supplement: Attachment — Submitted filename: review comments.docx [file pone.0262202.s002.docx]

This is a study reporting trends in admission rates, source of admission and diagnostic categories for babies admitted to a large tertiary newborn care unit in Southern China. Data have been derived from routine hospital data systems. Authors have used a time series analysis to compare two time periods: (i) pre-covid pandemic non pharmaceutical intervention implementation and (ii) covid pandemic non pharmaceutical interventions. Trends in hospital use, particularly those relating to newborns as a result of the pandemic have not frequently been reported. Clear drop in admission rates are demonstrated following the implementation of lockdown and other associated measures. Reductions were also seen in proportion of babies transferred in from neighbouring hospitals.

**Major comments**

These represent interesting descriptive data, however would be much richer if measures such as neonatal mortality, gestational age, birth weight and diagnosis of neonatal encephalopathy were included. It would be unusual for these measures, in particular mortality, not to be available from routine hospital data and therefore it seems odd not to have reported these. For instance the authors comment in the discussion that medical complexity and severity increased in those admitted in the second time period – but provide no data with which to show this. Tantalisingly they also comment that rates of therapeutic cooling increase but do not comment on diagnostic rates of neonatal encephalopathy (a sentinel marker of quality of perinatal maternal care). Please clearly state available measures from the HIS and why you have chosen the ones you have chosen.

Authors need to rewrite sections of the methods to describe more clearly the setting. They should not assume understanding or prior knowledge of this region, especially as lockdown and infection rates have been vastly different across the globe.

I think this paper could be improved enormously with inclusion of broader measures of newborn diagnosis and outcomes.

Methods need to set the scene much more clearly – for example, what is the population size covered by this unit (which from the description of newborn interventions provided would not be described as low resource). What is the usual care fatality rate for this unit? How premature are the babies who are cared for? What is the newborn birth rate within the hospital? It is not clear at all what admissions from outpatients refers to – please describe this more clearly. Anecdotally from my own work in Hong Kong a number of babies with prolonged jaundice and poor weight gain were admitted via outpatients to the NICU – not typical of UK settings. Is this similar in your hospital?

Methods shound be reported in accordance with the Strengthening the Reporting of Observational Studies in Epidemiology (STROBE) statement.

Also authors need to set the scene in the methods describing what policies were put in place by the local hospital systems due to COVID – for example, were hospitals told to reduce transfers? What were the pathways to admit from a referring hospital? Do you have isolation rooms etc? what lockdown/ NPIs were put in place and how did these change over time after the 24^th^? What are the staffing levels in the unit? How many ventilated beds/cots do you have? Did that change during the lockdown period?

Results comment around proportion of emergency and inter-hospital transfers increasing (lines 132) is confusing. Would be easier to understand if the methods included a brief description of where babies can be admitted from, eg. “babies can be admitted to this neonatal unit from 3 sources: labour ward/maternity care (inborn), outborn (transfers in from other hospitals) and outpatients (outborn).”

Univariate analysis appear to have been unnecessarily repeated – eg table 1 – chi2 should be conducted for one group of categorical outcomes rather than for each individual one. Please only present a single p value therefore for the group.

Would have been useful to present % increase or decrease by week of admission rates for example.

Results – major concern is exclusion of case fatality rate data and diagnosis of neonatal encephalopathy.

More detail can be given to jaundice- were these increases in pathological early jaundice, physiological jaundice or prolonged jaundice?

Discussion needs editing to explore data in more detail. For example what could the impact of this been on the wider health economy? did more babies die? What is the projected change in admissions like over time? When do you expect to reach previous levels? Or will you? Has the healthcare delivery in other settings/ home improved? Were some of the previous admissions inappropriate/unnecessary? More thought needs to be given to potential generalisability eg lines 201-202 – this is simply not true of the typical newborn population and likely related to the fact you are a tertiary surgical centre.

Discussion needs considerable rewriting to revisit some of the statements that are not evidenced through the data presented here.

Line 219 – I would argue that this is the most interesting data from this paper and least explored. As discussed above – what were the rates of neonatal encephalopathy diagnosis/ of deaths? Can you comment on reduced ventilated babies – is this because there were fewer premature babies (something reported in other countries) – can you test that within your data by looking at gestational age on admission?

Lines 223-224 – what is your evidence to back this statement up?> how have medical resources been more efficiently allocated? Might you want to continue this efficient allocation in the future (assuming you can evidence this statement). How has this allocation of resources impacted upon neonatal mortality and morbidity and on parental experience of healthcare? And on maternal outcomes? Were you able to measure and report on stillbirth rates for example?

Lines 224-225 – you don’t present any data to back this statement up in this paper. I have no doubt that has happened but it appears outkeeping with the data.

Lines 227-230 – you do not present any evidence to back this statement up at all.

Lines 231-232 – this is likely true, but how do your data help with this? Admission rates alone are not sufficient measure (and in fact one could argue an extremely poor measure) of efficacy of the healthcare system.

Lines 232-233 – this is also undoubtedly true – but how does this relate to your data?

Lines 238-239 – arguably this is a moderately well resourced unit – more detail needs ot be provided to allow the reader to compare resource of this unit to other settings.

Lines 241-243 – not really, you don’t show change in newborn behaviour.

Need to explore the what next? What additional data would be useful here with which to explore some of these hypotheses? For example from the wider health economy/ qualitative data from parents/ healthcare workers?

**Conclusion** – poorly written

**General comments**

Newborns arguably are unable to behave in any particular way – rather the healthcare systems and their parents/carers/ maternity services vary. Please rephrase “newborn healthcare behaviour” (used throughout the paper).

Ensure term pandemic rather than epidemic is used throughout

**Minor comments**

- Long and short title need to state location

Abstract

- background –needs to state where this study has been conducted
- methods – needs to include the time periods and which lockdown interventions and when
- methods - Define what the pre-COVID and COVID-impacted periods are. Seems pre-COVID period is very short
- conclusion – insufficient – needs to comment on fact that admissions reduced dramatically, these data only capture a small part of the picture – what happened to the non-admitted babies – did overall infant mortality rate increase for example?

Introduction

More information on background literature needs to be included here. For example a modelling study of this threat predicted between 250000-1.1 million extra neonatal deaths as a result of decreased service provision and access in LMICs. https://www.medrxiv.org/content/10.1101/2021.01.06.21249322v1

Methods:

Do HIS statistics capture all neonatal admissions for the region –and which region, how big, what population covered.

Statistical methods do not match

**Introduction**

Line 51 – “in” not “since”

Line 55 – “caused” not “causes”

Line 60 – consider abbreviation for NPI – non pharmaceutical interventions and then use this raterh than lockdown throughout

Line 63 “describing .. changes in newborn care” rather than “on the..”

Line 66 – see comment re phrase newborn medical behaviour being in appropriate for this age group

**Methods**

Line 71 – do you mean 10,000 babies per year over the last 5 years? Or 10,000 over the last 5 years? Unclear, please edit for sense.

Line 74 – no need for “besdies”

Line 91 – see comment re phrase newborn medical behaviour being in appropriate for this age group

Line 112 – I would not reloy on differentiating btween p values 0.05 or 0.01.

Methods- please describe how and when the diagnoses were given to the babies and by whom.

**Results**

Line 123 – replace visits with admissions

Lines 121-127 – please use phrase admissions per week (if this is what you are referring to) and edit this section for sense. For example – phrase “then it was reduced to a nadir of 108 people…” is not clear – do you mean it reduced to an admission rate of 108 babies/ week or that there were only 108 babies resident on the unit for that particular week?

figure 1 – please include dates on x axis, and also edit y axis to more accurately represent this measure (which I think is admissions per week)

figure 1 – annotate the figure to include what the dotted line indicates rather than just including this in the footnotes

line 132 – pandemic, not epidemic

line 132 – do not jump to causality here.

Lines 155-156 do not make sense

Lines 156-157 – when did a relatively high propotion of babies present with jaundice related conditions?

Figure 2 – please clarify and annotate as per figure 1

Discussion

Lines 177-178 edit for sense. Also highlight that these other cohorts do not include newborn data

Line 182 – pandemic not epidemic

Line 182 – edit to read admissions rather than entries

Line 195 – please include this detail in the methods

Line 206 – be more specific here between physiological/ pathological jaundice

Line 217 – how might you have measured degree of severity?

Line 218 – pandemic not epidemic
