# Supplementary material for: Impact of the COVID-19 pandemic on neonatal admissions in a tertiary children’s hospital in southwest China: An interrupted time-series study
Source: PLoS One. 2022 Jan 13;17(1):e0262202. doi: 10.1371/journal.pone.0262202 (PMC8758080; doi:10.1371/journal.pone.0262202)
Supplement: Attachment — Submitted filename: Response to Reviewers.docx [file pone.0262202.s003.docx]

**Point by Point Response to the Editor and Reviewers**

**Response to the comments of Editor**

**Comment 1：**A thorough language revision of the manuscript is needed to ensure clarity and consistency. Please also double-check any numbers and calculations presented

**Response: According to the reviewer’s suggestions, the manuscript has been polished by an English language editing company. We have also carefully checked the grammar errors in the revised manuscript (AJE).**

**Comment 2：**Please indicate the form of consent obtained (written/oral) or the reason that consent was not obtained

**Response: The oral consent was obtained prior to the preprint and initial submission of the manuscript.**

**Comment 3：**Consider including measures such as neonatal mortality, gestational age, birth weight and diagnosis of neonatal encephalopathy in the analysis; if not please state why not

**Response: We have added the description in the table1 "gestational age and birth weight" in Results section.No significant differences in gestational age or birth weight were observed between groups. The neonatal mortality rate increased by 0.09% during the COVID‐19 outbreak than the pre‐COVID‐19 period (Page12, Lines 189--191). Although the volume of admissions of newborns that suffering from neonatal encephalopathy was decreased, the results showed an elevated proportion of therapeutic hypothermia during NPIs (Page12, Lines 188--189).**

**Comment 4：**Methods should be reported in accordance with the Strengthening the Reporting of Observational Studies in Epidemiology (STROBE) statement

**Response: According to the reviewer’s suggestion, we have followed the STROBE guidelines for reporting observational studies (Page 4-7, Lines 69--145).**

**Comment 5：**discussion on the negative impact on infant outcomes is warranted

**Response: According to the reviewer’s suggestion, we have provided a deep discussion on the negative impact of the COVID-19 pandemic on newborn medical behaviors in the revised manuscript (Page14- 15, Lines 232--248).**

**Firstly, the results showed an elevated proportion of therapeutic hypothermia during NPIs. Some pregnant women reduced the frequency of antenatal visits due to the fear of contracting COVID-19.Therefore, some potential risk factors may not have been detected in a timely manner, leading to fetal distress and necessitating emergency delivery. Secondly,there was some evidence that the delay of accessing the tertiary care hospital by parents may have resulted in additional laboratory investigations, increases in the days of hospitalization, neonatal intensive care unit admission, and/or death.**

**Comment 6：**Please elaborate more on the limitations of the chosen analytical method e.g. seasonal considerations

**Response: To elaborate the limitation of the interrupted time series regression analyses, we have added the contents in the limitation section (Page15- 16, Lines 259--267).**

**Interrupted time series regression analyses are regarded as the strongest "quasi-experimental" approach and particularly useful when an RCT is infeasible. This finding has to be interpreted with caution because the study design did not investigate seasonal effects. Use of many treatments varies seasonally because of cyclic variations in the illnesses for which they are prescribed. Accounting for seasonally correlated errors usually requires at least 24 monthly data points. Interrupted time series regression analyses models we discussed assume a linear trend in the outcome within each segment. The assumption of linearity often may hold only over short intervals. Changes may follow non-linear patterns^1^.**

**Reference:**

1.Wagner AK, Soumerai SB, Zhang F, Ross-Degnan D. Segmented regression analysis of interrupted time series studies in medication use research. J Clin Pharm Ther. 2002; 27:299-309. https://doi.org/10.1046/j.1365-2710.2002.00430.x PMID: 12174032

**Replies to Reviewer 1**

**Comment 1:** These represent interesting descriptive data, however would be much richer if measures such as neonatal mortality, gestational age, birth weight and diagnosis of neonatal encephalopathy were included. It would be unusual for these measures, in particular mortality, not to be available from routine hospital data and therefore it seems odd not to have reported these. For instance the authors comment in the discussion that medical complexity and severity increased in those admitted in the second time period – but provide no data with which to show this. Tantalisingly they also comment that rates of therapeutic cooling increase but do not comment on diagnostic rates of neonatal encephalopathy (a sentinel marker of quality of perinatal maternal care). Please clearly state available measures from the HIS and why you have chosen the ones you have chosen.

**Response: We have added the description in the Table1 "gestational age and birth weight,neonatal encephalopathy, neonatal mortality" in Results section.**

**Comment 2**.Authors need to rewrite sections of the methods to describe more clearly the setting. They should not assume understanding or prior knowledge of this region, especially as lockdown and infection rates have been vastly different across the globe.

**Response: According to the reviewer’s suggestion, we have rewritten sections of the methods to describe more clearly the setting.**

**The Department of Neonatology of the Children's Hospital of Chongqing Medical University is a tertiary care hospital and the most extensive local neonate referral center in southwestern China. This facility offers a comprehensive range of services to the districts of Chongqing and neighboring provinces, including Guizhou, Sichuan, Yunnan, and Tibet, and serves approximately 350,000 babies in the associated area.**

**Comment 3:**I think this paper could be improved enormously with inclusion of broader measures of newborn diagnosis and outcomes.

**Response: We have added a newborn diagnosis in Results section(Neonatal encephalopathy ).**

**Neonatal encephalopathy (NE) is a clinically defined syndrome of disturbed neurologic function in an infant born at or beyond 35 weeks of gestation, manifested by a subnormal level of consciousness or seizures, and often accompanied by difficulty with initiating and maintaining respiration and depression of muscle tone and primitive reflexes****^1-3^****. The outcomes following neonatal encephalopathy include death and neurological disabilities such as cerebral palsy, epilepsy and cognitive impairment****^4^****^,^** **^5^****.**

**Reference:**

1.Executive summary: Neonatal encephalopathy and neurologic outcome, second edition. Report of the American College of Obstetricians and Gynecologists' Task Force on Neonatal Encephalopathy. Obstetrics and gynecology 2014; 123(4): 896-901.

2. Dixon B, Reis C, Ho W, Tang J, Zhang J. Neuroprotective Strategies after Neonatal Hypoxic Ischemic Encephalopathy. International journal of molecular sciences 2015; 16(9): 22368-401.

3. Edwards A, Brocklehurst P, Gunn A, et al. Neurological outcomes at 18 months of age after moderate hypothermia for perinatal hypoxic ischaemic encephalopathy: synthesis and meta-analysis of trial data. BMJ (Clinical research ed) 2010; 340: c363.

4. Hintz S, Barnes P, Bulas D, et al. Neuroimaging and neurodevelopmental outcome in extremely preterm infants. Pediatrics 2015; 135(1): e32-42.

5. Doyle L, Roberts G, Anderson P. Outcomes at age 2 years of infants < 28 weeks' gestational age born in Victoria in 2005. The Journal of pediatrics 2010; 156(1): 49-53.e1.

**Comment 4:**Methods need to set the scene much more clearly – for example, what is the population size covered by this unit (which from the description of newborn interventions provided would not be described as low resource). What is the usual care fatality rate for this unit? How premature are the babies who are cared for? What is the newborn birth rate within the hospital? It is not clear at all what admissions from outpatients refers to – please describe this more clearly. Anecdotally from my own work in Hong Kong a number of babies with prolonged jaundice and poor weight gain were admitted via outpatients to the NICU – not typical of UK settings. Is this similar in your hospital?

**Response: We agree and we have tried to mitigate this problem by fully rewrite sections of the methods.**

**This facility offers a comprehensive range of services to the districts of Chongqing and neighboring provinces, including Guizhou, Sichuan, Yunnan, and Tibet, and serves approximately 350,000 babies in the associated area. The Department of Neonatology has 310 beds and provides services that account for nearly 10,000 hospital admissions each year over the last 5 years.**

**Neonatal mortality rate 1.03% in Pre‐COVID period while neonatal mortality rate 1.12% in COVID impacted period.**

**In Pre-COVID period study, 1.65% of the newborn (n=201)had a gestational age less than 28 weeks,5.93% (n=705)had a gestational age of 28+0–31+6 weeks and 23% (n=2775)with a gestational age of 32+0–36+6 weeks.**

**In COVID impacted study,1.63% of the newborn (n=74)had a gestational age less than 28 weeks,5.48% (n=249) had a gestational age of 28+0–31+6 weeks and 23.30% (n=1056)with a gestational age of 32+0–36+6 weeks.**

**Babies can be admitted to this neonatal unit from 2 sources: outborn (transfer from other medical facilities and emergence) and outpatients (outborn).**

**The most common causes of outpatients included Neonatal pneumonia, pathological early jaundice and prolonged jaundice. Gestational age was near-term and full-term.**

**We recruited critically ill neonates from other medical facilities and emergence. Such as neonatal respiratory distress syndrome(NRDS), meconium aspiration syndrome(MAS), Hypoxic-ischemic encephalopathy(HIE), very low birth weight (<1500 g)/ low birth weight (1500–2499 g), preterm and so on.**

**Comment 5:**should be reported in accordance with the Strengthening the Reporting of Observational Studies in Epidemiology (STROBE) statement.

**Response:We have followed the STROBE guidelines for reporting observational studies(Page 4-7, Lines 69--145)..**

**Comment 6:**.Also authors need to set the scene in the methods describing what policies were put in place by the local hospital systems due to COVID – for example, were hospitals told to reduce transfers? What were the pathways to admit from a referring hospital? Do you have isolation rooms etc? what lockdown/ NPIs were put in place and how did these change over time after the 24th? What are the staffing levels in the unit? How many ventilated beds/cots do you have? Did that change during the lockdown period?

**Response: We have described the hospital systems policies in the methods.**

**The Chongqing government initiated the first-level response to major public health emergencies and the implementation of the NPIs on January 24, 2020. After the start of the NPIs, the hospital established a special isolation ward that was equipped with 8-16 beds, 5-8 trained nurses, and 2 pediatricians. The ward was equipped with a ventilator including invasive and noninvasive (CPAP and NIPPV) ventilation. During the pandemic, there was no requirement to reduce neonatal .**

**Comment 7:**Results comment around proportion of emergency and inter-hospital transfers increasing (lines 132) is confusing. Would be easier to understand if the methods included a brief description of where babies can be admitted from, eg. "babies can be admitted to this neonatal unit from 3 sources: labour ward/maternity care (inborn), outborn (transfers in from other hospitals) and outpatients (outborn)."

**Response: Thank the reviewer’s suggestion. Babies can be admitted to this neonatal unit from 2 sources: outborn (transfer from other medical facilities and emergence) and outpatients (outborn). We have included this description in the methods of the revised manuscript.**

**Comment 8:**Univariate analysis appear to have been unnecessarily repeated – eg table 1 – chi2 should be conducted for one group of categorical outcomes rather than for each individual one. Please only present a single p value therefore for the group.

**Response: As the reviewer suggested, we have amended the table in the revised maunuscript (Page 8, table 1).**

**Comment 9:**Would have been useful to present % increase or decrease by week of admission rates for example.

**Response: As suggested by the reviewer, we have presented the percent of increase or decrease by week of asmission rates in the revised manuscript.**

**The admissions per week of neonates dropped by 53%. The number of admissions continued to decline until it reached a nadir of 108 babies/week (Maximum number of hospital admissions 268 babies/week, dropped by 60 %).**

**Comment 10:**Results – major concern is exclusion of case fatality rate data and diagnosis of neonatal encephalopathy.

More detail can be given to jaundice- were these increases in pathological early jaundice, physiological jaundice or prolonged jaundice?

**Response: Fatality rate data and neonatal encephalopathy has been added to the Results section.**

**Jaundice refers to Pathological jaundice-related diseases(Such as : pathological jaundice;Neonatal hyperbilirubinemia; Hemolytic Disease of the Newborn;Glucose-6-phosphate dehydrogenase (G6PD) deficiency;bilirubin encephalopathy and so on).**

**Comment 11:**Discussion needs editing to explore data in more detail. For example what could the impact of this been on the wider health economy? did more babies die? What is the projected change in admissions like over time? When do you expect to reach previous levels? Or will you? Has the healthcare delivery in other settings/ home improved? Were some of the previous admissions inappropriate/unnecessary? More thought needs to be given to potential generalisability eg lines 201-202 – this is simply not true of the typical newborn population and likely related to the fact you are a tertiary surgical centre.

**Response: The results showed a slow recovery of increase in the admissions per week of the neonatal department. Nevertheless, more time is required to return to the pre-COVID period levels of admissions. After 1 year (January 24, 2021), the admissions per week of the neonatal has not recovered to pre-COVID period levels.**

**The neonatal mortality rate increased by 0.09% during the COVID‐19 outbreak compared with the pre‐COVID‐19 period (Table 1).**

**I agree fully with the reviewers comments. The possible reason may be overly liberal criteria for admitting patients in pre-COVID, some of which may not have been necessary. However, this COVID‐19 outbreak may indirectly reflect that the pandemic elevated the threshold for inpatient hospitalization.**

**This study was derived from a single tertiary care center with a retrospective design and a relatively small sample size. Therefore, it does not have strong universal validity, especially for primary health care.**

**We agree with the reviewer that the COVID-19 pandemic has had an unprecedented effect on health care and the economy, and this analysis will be carried out in the future elsewhere.**

**Comment 12:**Discussion needs considerable rewriting to revisit some of the statements that are not evidenced through the data presented here.
Line 219 – I would argue that this is the most interesting data from this paper and least explored. As discussed above – what were the rates of neonatal encephalopathy diagnosis/ of deaths? Can you comment on reduced ventilated babies – is this because there were fewer premature babies (something reported in other countries) – can you test that within your data by looking at gestational age on admission?
Lines 223-224 – what is your evidence to back this statement up?> how have medical resources been more efficiently allocated? Might you want to continue this efficient allocation in the future (assuming you can evidence this statement). How has this allocation of resources impacted upon neonatal mortality and morbidity and on parental experience of healthcare? And on maternal outcomes? Were you able to measure and report on stillbirth rates for example?
Lines 224-225 – you don't present any data to back this statement up in this paper. I have no doubt that has happened but it appears outkeeping with the data.
Lines 227-230 – you do not present any evidence to back this statement up at all.
Lines 231-232 – this is likely true, but how do your data help with this? Admission rates alone are not sufficient measure (and in fact one could argue an extremely poor measure) of efficacy of the healthcare system.
Lines 232-233 – this is also undoubtedly true – but how does this relate to your data?
Lines 238-239 – arguably this is a moderately well resourced unit – more detail needs ot be provided to allow the reader to compare resource of this unit to other settings.
Lines 241-243 – not really, you don't show change in newborn behaviour.
Need to explore the what next? What additional data would be useful here with which to explore some of these hypotheses? For example from the wider health economy/ qualitative data from parents/ healthcare workers?

**Response: Thank you for your advice. After a detailed discussion of the reviewer's suggestion, we are in agreement with your suggestion and have removed it.**

**Comment 13:**Conclusion – poorly written

**Response: According to your suggestion,the Conclusion section was rewritten(Page 16, Lines 270-277).**

**Comment 14:**General comments
Newborns arguably are unable to behave in any particular way – rather the healthcare systems and their parents/carers/ maternity services vary. Please rephrase "newborn healthcare behaviour" (used throughout the paper).
Ensure term pandemic rather than epidemic is used throughout

**Response: It should be “newborn healthcare behaviour and pandemic” and appropriate correction has been made in the revised manuscript.**

**Minor comments**
**Comment 1:**Long and short title need to state location

**Response: COVID-19 pandemic on newborn medical behaviors**

**Comment 2:**.Abstract
• background –needs to state where this study has been conducted
• methods – needs to include the time periods and which lockdown interventions and when
• methods - Define what the pre-COVID and COVID-impacted periods are. Seems pre-COVID period is very short
• conclusion – insufficient – needs to comment on fact that admissions reduced dramatically, these data only capture a small part of the picture – what happened to the non-admitted babies – did overall infant mortality rate increase for example?

**Response:We thank the reviewer for the very helpful suggestions and advice. We have rewritten the part of the Abstract the reviewer found problematic.**

**Comment 3:**.Introduction
More information on background literature needs to be included here. For example a modelling study of this threat predicted between 250000-1.1 million extra neonatal deaths as a result of decreased service provision and access in LMICs. https://www.medrxiv.org/content/10.1101/2021.01.06.21249322v1
**Response: While newborns are at low risk for severe disease and death from COVID-19, any impacts on their health outcomes will likely be attributable to the indirect effects of the pandemic on health systems^1,2^.**

**Reference:**

1.Liguoro I, Pilotto C, Bonanni M, Ferrari ME, Pusiol A, Nocerino A, et al. SARS-COV-2 infection in children and newborns: a systematic review. Eur J Pediatr. 2020; 179:1029-46. https://doi.org/10.1007/s00431-020-03684-7 PMID: 32424745

2. Castagnoli R, Votto M, Licari A, Brambilla I, Bruno R, Perlini S, et al. Severe Acute Respiratory Syndrome Coronavirus 2 (SARS-CoV-2) Infection in Children and Adolescents: A Systematic Review. JAMA Pediatr. 2020; 174:882-9. https://doi.org/10.1001/jamapediatrics.2020.1467 PMID: 32320004

**Comment 4:**Introduction
Line 51 – "in" not "since"
Line 55 – "caused" not "causes"
Line 60 – consider abbreviation for NPI – non pharmaceutical interventions and then use this rater than lockdown throughout
Line 63 "describing .. changes in newborn care" rather than "on the.."
Line 66 – see comment re phrase newborn medical behaviour being in appropriate for this age group
Methods
Line 71 – do you mean 10,000 babies per year over the last 5 years? Or 10,000 over the last 5 years? Unclear, please edit for sense.
Line 74 – no need for "besdies"
Line 91 – see comment rephrase newborn medical behaviour being in appropriate for this age group
Line 112 – I would not reloy on differentiating btween p values 0.05 or 0.01.
Methods- please describe how and when the diagnoses were given to the babies and by whom.

**Response: Correction has been made in the revised manuscript.**

**Comment 5:**Results
Line 123 – replace visits with admissions
Lines 121-127 – please use phrase admissions per week (if this is what you are referring to) and edit this section for sense. For example – phrase "then it was reduced to a nadir of 108 people…" is not clear – do you mean it reduced to an admission rate of 108 babies/ week or that there were only 108 babies resident on the unit for that particular week?
figure 1 – please include dates on x axis, and also edit y axis to more accurately represent this measure (which I think is admissions per week)
figure 1 – annotate the figure to include what the dotted line indicates rather than just including this in the footnotes
line 132 – pandemic, not epidemic
line 132 – do not jump to causality here.
Lines 155-156 do not make sense
Lines 156-157 –when did a relatively high propotion of babies present with jaundice related conditions?

Figure 2 – please clarify and annotate as per figure 1

**Response: Correction has been made in the revised manuscript.**

**Comment 6:**Discussion
Lines 177-178 edit for sense. Also highlight that these other cohorts do not include newborn data
Line 182 – pandemic not epidemic
Line 182 – edit to read admissions rather than entries
Line 195 – please include this detail in the methods
Line 206 – be more specific here between physiological/ pathological jaundice
Line 217 – how might you have measured degree of severity?
Line 218 – pandemic not epidemic

**Response:This comment has been accepted. The correction has been made in the revised manuscript.**

**Replies to Reviewer 2**

**Comment 1:**While the study is statistically impeccable and the writing is very clear, more discussion of the negative impact on infant outcomes seems warranted. Similarly, if there has not been a commensurate increase in negative outcomes does that suggest many NICU admissions are unnecessary? The decrease in volume by itself is not that interesting. One approach might be a parallel ITS of negative outcomes with the same interruptions specified.

**Response: We have analysis the negative impact of the COVID-19 pandemic on newborn medical behaviors in the discussion section (Page 14-15, Lines 232--248).**

**Replies to Reviewer 3**

**Comment 1:**In the abstract the authors stated that "Overall, 13,540 infants were admitted to the NICU during the pre-COVID period (n= 12082) and COVID-impacted period (n=4558)." The figures provided in line 34 – 35 do not add up and the statement is not clear on the disparity between the 13,540 and the figures provided in parentheses. It is also not clear what NICU means at this stage.

**Response:We are very sorry for our incorrect writing that 'Overall, 13,540 infants were admitted to the NICU', according to your suggestion, we have re-written this part in the abstract section.**

**Overall, 16,640 infants were admitted to the neonatology department during the pre-COVID period (n = 12,082) and the COVID-impacted period (n =4,558).**

**Comment 2:**In line 64 it seems that the impact of lockdown measures ( such as curfew, opening and closing hours of business, who and where one could travel to ) on neonatal admissions and disease spectrum are to be studied. However, that seems not to be the case. The analysis and results presented seem to have studied if there is changes in neonatal admission and diseases spectrum pre-COIVD and during the COVID period. Or are the authors referring to the period of lockdown as the "lockdown measures"? This should be clarified in the manuscript.

**Response: The article was to detect changes in the levels and trends of hospital admissions and disease spectra** **before and after the period of lockdown measures and assess the impact of the control measures.**

**Comment 3:**In line 77 it was stated the "clinical and demographics information were collected…" while in line 78 though attempt was made to list the clinical information but the lists provided in line 79 does not differentiate between clinical and demographic information.

**Response:Correction has been made in the revised manuscript.**

**The variables selected to describe the demographic and clinical information of the cases were** **the volume of admissions and neonate transport, sex, age** **(days), admission, length of hospital stay (days), treatment, neonatal mortality, gestational age (weeks + days), birth weight (gram) and disease spectra.**

**Comment 4:**The authors should consider formatting the date on line 84 and 85 to commonly used time format for example (yyyy/m/d – yyyy/m/d).

**Response: The format of the date has been changed in the revised manuscript.**

**Comment 5:**In the manuscript in line 87 to 91 the outcomes and dependent variables were not clearly stated. Clarity is needed for the readers.

**Response: The outcome variable was the weekly number of cause-specific hospital admissions. The dependent variable was time, intervention and Post-slope.**

**Comment 6:**The use of interrupted time-series and the choice of break point seem appropriate. However, the authors should consider a change point analysis to detect a change in the data generating process. This might as well provide the basis for choosing or confirming the choice of the break point used in this analysis. In addition, time series analyses may be confounded by seasonal effects and there is no discussion in the manuscript if it is an issues and how it was addressed.

**Response: To elaborate the limitation of the interrupted time series regression analyses, we added the contents in the limitation section (Page15- 16, Lines 259--267).**

**We agree with the reviewer that change point analysis to detect a change in the data generating process, and this analysis will be carried out in the future elsewhere.**

**Comment 7:**In line 108, the mean should be reported with the standard deviation or the median and the interquartile range.
In lines 134 – 138 of the manuscript, the medians and not the means were reported. Please ensure consistency with line 108.

**Response:We apologize for this mistake,the correction has been made in the revised manuscript(Page 7, line 138).**

**Comment 8:**In line 139 table 1. The table needs formatting to ensure clarity. Are the values for neonatal transport part of the Admission group ( the values under the category admission did add up to the total). It is not obvious from the table, which values neonatal transport, and Treatment falls under or how they each sum to the total?

**Response: As reviewer suggested,the table have been amended to make it as simple as possible to understand(Page 8, table 1).**

**Comment 9:**In line 181 "… decreased by -68% and -45%,…" should be corrected to "… decreased by 68% and 45%,…"

**Response: Appropriate correction has been made in the revised manuscript(Page 13, line 201**

**).**

**Comment 10:**Inline 181 do the authors mean "… slowly improving…."? The sentence should be revised for easy understanding.

**Response: With the abatement of the pandemic, the neonatal admission volume slowly recovered.**

Special thanks to you for your good comments.

We tried our best to improve the manuscript and made some changed in the manuscript. These changes are marked in red in revised paper.

We appreciate for editors' and reviewers' warm work earnestly, and hope that the correction will meet with approval.

Once again, thank you very much for your comments and suggestions.

Best regards

Sincerely

Corresponding author: Zi-Yu Hua

E-mail: [h_ziyu@126.com](mailto:h_ziyu@126.com)
