# Supplementary material for: Impact of the COVID-19 pandemic on neonatal admissions in a tertiary children’s hospital in southwest China: An interrupted time-series study
Source: PLoS One. 2022 Jan 13;17(1):e0262202. doi: 10.1371/journal.pone.0262202 (PMC8758080; doi:10.1371/journal.pone.0262202)
Supplement: Attachment — Submitted filename: Response to Reviewers.docx [file pone.0262202.s004.docx]

**Point by Point Response to the Editor and Reviewers**

**Response to the comments of Editor**

**Comment 1：**Please answer, all the comments of the reviewers. As they highlight, there are several points that need to be addressed mainly in the data description and results interpretation.  The results (for example, table 1) would be easier to be interpreted if presented as rate per week. Otherwise, the values by disease groups, treatment, mortality and others produce confusion. Besides, to observe the variation in neonatal mortality the change in case fatality rate/ week over time should be added.

**Response: We agree and we have tried to mitigate this problem by fully rewrite sections of the results( table 1) .We have added the description in the table1 "fatality rate/ week " in Results section****(Page 8, Lines 169).**

**Replies to Reviewer 1**

**Comment 1:**For example, they should look to describe change in case fatality rate/ week over time to show how that varies before and after introduction of NPIs. Of note, the figures show drops in admissions from all causes, with recovery only apparent from pathological jaundice.

**Response:****According to the reviewer’s suggestion, We have added the description in the table1 "fatality rate/ week " in Results section(Page 8, Lines 169).**

**There was an evident decrease in the volume of admissions/week for all disease spectra after the intervention. Additionally, there was a statistically significant decrease in the number of patients with pathological jaundice-related conditions(*p*<0.05)(Table 2 and Figure 2).**

**However, their recovery trend was faster than that other disorders (*p*<0.05), therefore relatively speaking the proportion of admissions attributable to jaundice increased.**

**Other comments:**

**Comment 2.**Please state where this study is conducted in both the – not addressed.

**Response: Correction has been made in the revised manuscript.**

**long title: Impact of the COVID-19 Pandemic on neonatal admissions in a tertiary Children's Hospital in Southwest China: an interrupted time-series study.**

**short title:COVID-19 pandemic affecting neonatal admissions in southwest China**

**Comment 3:**Please rephrase “newborn medical behaviours” throughout (not addressed)

**Response: It should be“health-seeking behavior of parents/caregivers.”and appropriate correction has been made in the revised manuscript.**

**Abstract**

**Comment 4:**Background – state “China” in location as many will not know where Chongqing Medical University is.

**Response: We thank the reviewer for the very helpful suggestions and advice. We have rewritten the part of the Abstract the reviewer found problematic.**

**This study was conducted to investigate the impact of lockdown measures in a tertiary Children's Hospital in southwest China, which might be used to predict long-term effects related to health-seeking behavior of parents/caregivers****(Page 1, Lines 20-22).**

**Comment 5:**Results section appear to be contradictory and confusing – please edit for sense. This section does not make sense “There was no decline in the volume of admissions of newborns suffering from respiratory system diseases, infectious diseases, and gastrointestinal diseases immediately after the COVID-impacted period (p =0.08, p =0.11, p =0.06, respectively). There was an immediate decline in the volume of patients complaining about pathological jaundice-related disease conditions” – however table 1 and figures show casing the data suggests falls in all of these admissions.

**Response: We thank the reviewer for the very helpful suggestions and advice. We have rewritten the part of the Abstract the reviewer found problematic(Page 2, Lines 35-43).**

**The average weekly admission rates of 220/week pre COVID and 147/week post COVID. There was an evident decrease in the volume of admissions for all disease spectra after the intervention, whereas the decrease of patients complaining about pathological jaundice-related conditions was statistically significant (*p*<0.05). In the COVID-impacted period, the percentage of patients who suffered from respiratory system diseases, neonatal encephalopathy, and infectious diseases decreased, while the percentage of pathological jaundice-related conditions and gastrointestinal system diseases increased. The neonatal mortality rate increased by 9‰ during the COVID-19 outbreak compared with the pre-COVID period.**

**Setting**

**Comment 6:**It appears this neonatal unit does not accept direct referrals from a maternity unit/ labour ward? Is this correct? Please clarify and add this detail to the methods. What does the emergency category pertain to?

**Response: According to the reviewer’s suggestion, we have rewritten sections of the methods to describe more clearly the setting.(Page 4, Lines 90-94).**

**Dedicated transport teams are staffed separately from NICU personnel specifically for the purpose of transport of patients to and from the hospital. These personnel do not have patient assignments. Our neonatal unit receives direct referrals from the labour ward or Level I or II neonatal care services using the transport system.**

**"Emergency" means that critically ill patients were transferred and admitted into our neonatal unit directly via outpatients.**

**Comment 7:**What proportion of the admissions are usually from out of province?

**Response:According to the reviewer’s suggestion, We have added the description in the table1 "Proportion of the admissions are usually from out of province " in Results section(Page 8, Lines 169).**

|  | **Pre‐COVID** | **COVID impacted** | ***p* value** |
| --- | --- | --- | --- |
| **Proportion of the admissions are usually from out of province，n (%)** | **2225(18.42)** | **702(15.41)** | ***p*<0.05** |

**Results**

**Comment 8:**“The patients seeking consultations in the COVID-impacted period were younger than those seeking consultations in the pre-COVID period 159 (median age 5 days vs. 6 days, p<0.05).” – this is likely because those admitted via outpatients were reduced – if you compare against age in those admitted from interhospital transfers only is there a difference in age?

**Response: I agree with these concerns, and feel that - age is associated with a higher percentage of in outpatients(outborn).**

**Comment 9:** Where is the p value for the mortality rate in table 1? Please present rate not percentage for a mortality rate (typically presented as case fatality rate of X babies per 1000 admitted babies) – here table 1 suggests a change from 10.3 per 1000 to 11.2 per 1000 – a 10% increase in mortality (not a 0.09% increase). Please clarify this on page 26 line 189-190.

**Response:**

| Average rate of weekly Fatality | Pre‐COVID | COVID impacted | p value |
| --- | --- | --- | --- |
| n (‰) | 2.3（10.3） | 1.6（11.2） | p<0.05 |

**NMRs increased by 9‰ during the COVID‐19 outbreak compared with the pre-COVID. However, no statistical differences were found between NMRs (*p*=0.66)****(Page 13, Lines 193-194).**

**Comment 10:**Data should be presented as weekly rates – for example in the text include average weekly admission rates of 220/week pre COVID and 147/week post COVID; average weekly admissions for respiratory disorders pre and post decreased from 76/wk to 48/wk; admissions for neonatal encephalopathy from 4/wk to 2.5/wk – of those admitted with NE – 23% required therapeutic hypothermia compared to 56% post covid – suggesting those babes were sicker on admission.

**Response:** **We agree and we have tried to mitigate this problem by fully rewrite sections of the results( table 1) .**

**Comment 11:**Weekly rates of pathological jaundice admissions dropped from 59/week to 46/week – but that drop was not as substantial as other disorders, therefore relatively speaking the proportion of admissions attributable to jaundice increased, but the absolute number reduced

**Response: We agree and we have tried to mitigate this problem by fully rewrite sections of the results(Page 12, Lines 184-188) .**

**There was an evident decrease in the volume of admissions/week for all disease spectra after the intervention. Additionally, there was a statistically significant decrease in the number of patients with pathological jaundice-related conditions(*p*<0.05)(Table 2 and Figure 2).**

**However, their recovery trend was faster than that other disorders (*p*<0.05), therefore relatively speaking the proportion of admissions attributable to jaundice increased.**

**Discussion**

**Comment 12:** Needs discussion on change in mortality rate
**Response:According to the reviewer’s suggestion, we have provided a deep discussion on change in mortality rate in the revised manuscript (Page15- 16, Lines 246--267).**

**The data suggested that neonatal death might be increased in COVID-impacted period. Preterm birth is the leading cause of neonatal mortality and morbidity in both high- and low-income countries.No significant differences were observed between groups in gestational age. Therefore, this increase in mortality rate appears to be driven by other factors and is not likely to be explained by preterm birth. It is more likely that changes in illness severity, health-seeking behavior of parents/caregivers, and inadequate medical resources factors.**

**The admissions for neonatal encephalopathy from 4/wk to 2.5/wk–of those admitted with NE–23% required therapeutic hypothermia compared to 56% post covid–suggesting those babes were sicker on admission. The neonatal period is classified as early (the first 7 days) and late (the remaining 21 days) neonatal period, and majority neonatal mortality occur in the early neonatal period. The proportion of children aged ≤7 days old was higher in the COVID-impacted period(54.7% pre-COVID and 58.8% post-COVID).In China, the implementation of NPIs, also resulting in severe restrictions affecting the health-seeking behavior of parents/caregivers. There was concern that** **parents/ caregivers stay at home to care for her baby with progressive disease rather than come to the hospital where the risk of contracting COVID-19 was perceived to be higher. The Department of Neonatology has insufficient critical care staff during COVID-impacted period. It is possible that reduced capacity of healthcare services to provide timely diagnoses and treatment. Reassuringly, these mortality analyses found no statistical differences (*p*=0.66). These results should be interpreted with caution, and additional studies are needed to clarify whether our observations represent a significantly higher mortality rate adjusted for disease severity or whether they merely reflect increased primary care management of less severe conditions.**

**Comment 13:**This statement “This is partly because gastrointestinal disorders are relatively more common in newborns due to inborn anomalies (e.g., megacolon, esophageal atresia, pyloric hypertrophic obstruction)[19]” is not correct. It may be that in your surgical unit these disorders are relatively more common, however in the typical neonatal population these are most certainly NOT more common than respiratory disorders/ infections.

**Response: We agree and we have tried to mitigate this problem by fully rewrite sections of the discussion (Page14, Lines 220-225).**

**However, the proportion of gastrointestinal diseases and surgeries were unaffected by these measures (*p*=0.21 and *p*=0.10). This is partly because gastrointestinal disorders, especially anomalies, are relatively more common in our tertiary care hospital (e.g., megacolon, esophageal atresia, pyloric hypertrophic obstruction). Some of these diseases require surgical intervention in the neonatal period. As one of the regional neonate emergency care providers and the most extensive local neonate referral center, only the children's hospital could perform a sequence of operations.**

**Comment 14:**This statement is not true “Almost all newborns develop pathological hyperbilirubinemia[20, 21]” furthermore references 20 and 21 relate to clinical assessment of jaundice rather than rates of pathological jaundice.

**Response: Neonatal hyperbilirubinemia is a common condition, affecting more than 60% of full-term and 80% of preterm infants [****19, 20, 21].**

19.Riordan SM, Bittel DC, Le Pichon JB, Gazzin S, Tiribelli C, Watchko JF, et al. A Hypothesis for Using Pathway Genetic Load Analysis for Understanding Complex Outcomes in Bilirubin Encephalopathy. Front Neurosci. 2016; 10:376. https://doi.org/10.3389/fnins.2016.00376 PMID: 27587993

20.Hegyi T, Kleinfeld A, Huber A, Weinberger B, Memon N, Shih W, et al. Unbound bilirubin measurements by a novel probe in preterm infants. J Matern Fetal Neonatal Med. 2019; 32:2721-6. https://doi.org/10.1080/14767058.2018.1448380 PMID: 29504491

21.Gazzin S, Dal Ben M, Montrone M, Jayanti S, Lorenzon A, Bramante A, et al. Curcumin Prevents Cerebellar Hypoplasia and Restores the Behavior in Hyperbilirubinemic Gunn Rat by a Pleiotropic Effect on the Molecular Effectors of Brain Damage. Int J Mol Sci. 2020; 22. https://doi.org/10.3390/ijms22010299 PMID: 33396688

**Comment 15:**Need to consider impact on entire health economy – eg did more mother’s deliver at home? What was the change in stillbirth rate? The change in mortality rates in the secondary care units? The discussion presents a far too simplistic discussion around potential explanations for the changes in rates and diagnosis documented.

**Response: We agree with the reviewer that the COVID-19 Pandemic has drastically impacted on entire health economys, and this analysis will be carried out in the future elsewhere.**

**Finally, The COVID-19 Pandemic has drastically impacted on entire health economy. For instance, there are potential barriers to accessing obstetric and neonatal health care services, barriers to good healthcare-seeking behavior of parents/ caregivers, transportation barriers, and parents/ caregivers’ financial barriers(housing instability, food insecurity). Further research should be conducted to elaborate on this aspect(Page17, Lines 220-225).**

**Comment 16:** Discussion of change in therapeutic hypothermia focuses entirely on antenatal access – by mothers – not at all on availability of these services due to health service changes nor to potential discussion of quality of care available during the perinatal period – this needs to be addressed as it does not adequately address potential factors involved in resultant NE, but instead focuses on maternal factors in their behaviour of accessing health services.

**Response: We agree and we have tried to mitigate this problem by fully rewrite sections of the discussion (Page15, Lines 237-245).**

**One probable reason for such unacceptable poor quality of maternity care and poor neonatal health outcome is changes in accessing emergency obstetric and /or neonatal cares which include delays in reaching health facilities (delays at the journey) and delays in receiving appropriate care (delays at the health facilities).**

Comment 17:Lines 242-245 – the data don’t reflect this. Too much weight is placed on parent behaviours.

**Response: Thank you for your advice. After a detailed discussion of the reviewer's suggestion, we are in agreement with your suggestion and have removed it.**

**Comment 18:**Limitations are not sufficiently well described – of note this is a surgical tertiary referral unit that doesn’t appear (from the description) to admit directly from maternity services – extremely unusual for most neonatal units across the globe.

**Response: According to the reviewer’s suggestion, we have rewritten sections of the methods to describe more clearly the setting.(Page 4, Lines 90-94).**

**Replies to Reviewer 3**

**Comment 1:**However, I have minor suggestions that might be useful for both the author and research community.In line 144 – 145, the author cited the R software, from the citation (I assume that the package used were R based package), if the packages are not “R base” the author should fully cite the packages if not shipped as part of R base functions. The citation of the packages acknowledges the effort and time spent by the people that created these tools and help in reproducibility of the results.
All citation should also appear in the reference list.
For example, using and citing RStudio and lme4 package assuming both are the 18th and 19th cited materials:
All computations were conducted in RStudios 1.0.44 (18) … with lme4 v.xxx (19)…. While in the reference list the citation for lme4 should appear as:
Douglas Bates, Martin Maechler, Ben Bolker, Steve Walker (2015). Fitting Linear Mixed-Effects Models Using lme4. Journal of Statistical Software, 67(1), 1-48. doi:10.18637/jss.v067.i01.
R Core TEAM(2014). R. A language and environment for statistical computing. R Foundation for Statistical Computing, Vienna, Austria. URL http://www.R-project.org/

**Response:Correction has been made in the revised manuscript.**

**Comment 2:**Table 1, in line 165 need a little adjustment. The section for weeks + day, require addition information. For instance 201(1.65), does it represent mean(sd) or median(IQR). Please let it be consistent like the section on Birth weight(gram), median(IQR), and the section on Age which has median (IQR) etc.

**Response: We apologize for this mistake,the correction has been made in the revised manuscript(Page 8, Lines 169).**

Special thanks to you for your good comments.

We tried our best to improve the manuscript and made some changed in the manuscript. These changes are marked in red in revised paper.

We appreciate for editors' and reviewers' warm work earnestly, and hope that the correction will meet with approval.

Once again, thank you very much for your comments and suggestions.

Best regards

Sincerely

Corresponding author: Zi-Yu Hua

E-mail: [h_ziyu@126.com](mailto:h_ziyu@126.com)
