# Supplementary material for: Impact of the COVID-19 pandemic on neonatal admissions in a tertiary children’s hospital in southwest China: An interrupted time-series study
Source: PLoS One. 2022 Jan 13;17(1):e0262202. doi: 10.1371/journal.pone.0262202 (PMC8758080; doi:10.1371/journal.pone.0262202)
Supplement: Attachment — Submitted filename: Response to Reviewers.docx [file pone.0262202.s005.docx]

**Point by Point Response to the Editor and Reviewers**

**Response to the comments of Editor**

**Comment 1：**Please review your reference list to ensure that it is complete and correct. If you have cited papers that have been retracted, please include the rationale for doing so in the manuscript text, or remove these references and replace them with relevant current references. Any changes to the reference list should be mentioned in the rebuttal letter that accompanies your revised manuscript. If you need to cite a retracted article, indicate the article’s retracted status in the References list and also include a citation and full reference for the retraction notice.

**Response: According to the reviewer’s suggestion, reference list is complete and correct.**

**Replies to Reviewer 1**

**Comment 1:** please ensure the phrase "COVID-impacted period" is used throughout to describe the second time period. In places (eg abstract methods and discussion) it is described as "after covid" or post-covid.

**Response:****According to the reviewer’s suggestion, correction has been made in the revised manuscript.**

**Replies to Reviewer 3**

**Comment 1:**Lines 250 – 251 “It is more likely that changes in illness severity, health-seeking 251 behavior of parents/caregivers, and inadequate medical resources factors.” , seems incomplete.

**Response: We thank the reviewer for the very helpful suggestions and advice. We have rewritten the part of the discussion the reviewer found problematic(Page 15, Lines 251-253 ).**

**It is more likely that changes in illness severity, health-seeking behavior of parents/caregivers, and inadequate medical resources factors result in this phenomenon.**

**Comment 2:**Line 124 – stated that is “experiment was designed” or do the author meant that “this study was conducted…”, because I don’t think an experiment was designed in the real sense of it.

**Response: We thank the reviewer for the very helpful suggestions and advice. We have rewritten the part of the methods the reviewer found problematic(Page6 , Lines 124-125).**

**Second, this study was conducted to evaluate whether the health-seeking behavior of parents/caregivers was influenced by pandemic factors.**

**Comment 3:**Lines 253, 275, 262 “…post COVID…” or do you mean COVID-impacted period ?

line 155 “…. 147/week post-COVID”, do the authors meant COVID-period ?

**Response: According to the reviewer’s suggestion, correction has been made in the revised manuscript.**

Special thanks to you for your good comments.

We tried our best to improve the manuscript and made some changed in the manuscript. These changes are marked in red in revised paper.

We appreciate for editors' and reviewers' warm work earnestly, and hope that the correction will meet with approval.

Once again, thank you very much for your comments and suggestions.

Best regards

Sincerely

Corresponding author: Zi-Yu Hua

E-mail: [h_ziyu@126.com](mailto:h_ziyu@126.com)
